# Autistic Disorders and Schizophrenia: Related or Remote? An Anatomical Likelihood Estimation

**DOI:** 10.1371/journal.pone.0012233

**Published:** 2010-08-18

**Authors:** Charlton Cheung, Kevin Yu, Germaine Fung, Meikei Leung, Clive Wong, Qi Li, Pak Sham, Siew Chua, Gráinne McAlonan

**Affiliations:** 1 Department of Psychiatry, Li Ka Shing Faculty of Medicine, The University of Hong Kong, Hong Kong, Hong Kong; 2 Centre for Reproduction Development and Growth, The University of Hong Kong, Hong Kong, Hong Kong; 3 State Key Laboratory for Brain and Cognitive Sciences, The University of Hong Kong, Hong Kong, Hong Kong; The University of Hong Kong, China

## Abstract

Shared genetic and environmental risk factors have been identified for autistic spectrum disorders (ASD) and schizophrenia. Social interaction, communication, emotion processing, sensorimotor gating and executive function are disrupted in both, stimulating debate about whether these are related conditions. Brain imaging studies constitute an informative and expanding resource to determine whether brain structural phenotype of these disorders is distinct or overlapping. We aimed to synthesize existing datasets characterizing ASD and schizophrenia within a common framework, to quantify their structural similarities. In a novel modification of Anatomical Likelihood Estimation (ALE), 313 foci were extracted from 25 voxel-based studies comprising 660 participants (308 ASD, 352 first-episode schizophrenia) and 801 controls. The results revealed that, compared to controls, lower grey matter volumes within limbic-striato-thalamic circuitry were common to ASD and schizophrenia. Unique features of each disorder included lower grey matter volume in amygdala, caudate, frontal and medial gyrus for schizophrenia and putamen for autism. Thus, in terms of brain volumetrics, ASD and schizophrenia have a clear degree of overlap that may reflect shared etiological mechanisms. However, the distinctive neuroanatomy also mapped in each condition raises the question about how this is arrived in the context of common etiological pressures.

## Introduction

Autistic spectrum disorders (ASD; comprising autism, high-functioning autism, and Asperger's syndrome) and schizophrenia have a substantial number of features in common. People with autism, have a strong family history of schizophrenia and bipolar disorder [1], [2], [3] and may have alterations in the same set of genes [4], [5]. Other aetiological factors such as maternal infection [6], [7], copy number variants in genetic structure [8] and maternal vitamin D deficiency during pregnancy [9], [10] have all been associated with increased risk of both disorders. Consistent with shared aetiological factors, ASD and schizophrenia share phenotypic characteristics. Asperger's syndrome is associated with higher scores on measures of paranoia than is typical [11] and individuals with autism may even suffer from psychosis [12]. ‘Negative’ symptoms reminiscent of schizophrenia are recognized in people with Asperger's syndrome and these partly respond to the antipsychotic risperidone [13]. Social interaction, communication, emotion processing and executive function abilities are disrupted by both conditions. The two conditions involve unusual responsiveness to the environment [14] and impaired stimulus filtering, which can be measured by a failure of sensorimotor gating in the prepulse inhibition of startle paradigm [15], [16], [17], [18]. Indeed, autism was originally referred to as a ‘schizophrenic syndrome of childhood' or ‘childhood psychosis’, and has been suggested to lie on the same spectrum as schizophrenia [19]. However the extent to which there is a common or distinctive brain substrate in ASD and schizophrenia has not been definitively quantified.

Resisting this position is an influential hypothesis recently proposed by Crespi and Badcock (2008). In their conceptualization, autism reflects a bias towards paternally expressed genes, brain overgrowth and underdevelopment of social brain systems. Schizophrenia, on the other hand, is said to involve maternally expressed genes, brain undergrowth and maladaptive ‘hyper-development’ of social systems. These two disorders therefore have abnormalities in the same set of traits but are ‘diametrically’ opposite, with opposing phenotypes. Thus the field is ripe for investigation of shared or unique characteristics of these two disorders, with the hope that this can inform the search for aetiological mechanisms driving neurodevelopmental dysfunction as well as possible fresh approaches to each condition.

The explosion in brain imaging studies over the past decade has greatly expanded our knowledge of brain biology in autistic disorders and schizophrenia. However, with the exception of one recent study which looked at autism and psychosis [12], MRI studies have focused on either ASD or schizophrenia exclusively, and there has been no direct test of brain structural similarities in these conditions. Therefore, the aim of the present study is to synthesize multiple imaging datasets addressing ASD and schizophrenia within a common framework for a detailed exploration. Consolidation of large imaging datasets is now possible using meta-analytic techniques including ‘Anatomical/Activation Likelihood Estimation’ (ALE) approaches [20], [21]. ALE merges datasets generated by voxel-based brain imaging studies which explore every ‘volume-element’ or voxel throughout the whole brain space. ALE allows these detailed results to be summarized, thereby identifying brain regions most consistently reported in the majority of studies. We have previously used ALE to map the brain differences underpinning progression of illness, for example from high risk through first episode and chronic schizophrenia [22] and the effect of drug treatment on brain morpholology in schizophrenia [23]. However the potential for an ALE analyses beyond comparison of single conditions with healthy control groups has not been fully realized.

The initial step of ALE is to generate a Gaussian probability distribution around the peak or central coordinates of significant foci reported in voxel-based studies. The probability that any given voxel is involved in the disorder(s) can be estimated from this whole brain ‘likelihood’ map. ALE retains foci that are close in proximity (regions most consistently reported across studies) to generate resultant or summary 3D clusters. The novelty of our current approach is to combine datasets for joint entry into a single analysis, therefore the resultant ALE map will contain clusters comprising foci from one or other or both conditions, and the contribution each disorder made to every resultant cluster can be estimated. This should help to clarify the extent to which ASD and schizophrenia cause similar brain structural phenotype. The prediction is that there will be considerable overlap across these conditions, a result that will point towards potentially common causal mechanisms.

## Methods

### Data Search

A search was carried out using PubMed, Scopus, and PsycINFO databases with the keywords including: autism, high functioning autism, Asperger, schizophrenia, first-episode, psychosis, MRI, voxel, VBM, and SPM (statistical parametric mapping). A branch search was conducted from the retrieved studies, and also existing meta-analysis studies on autistic spectrum disorders and schizophrenia. Schizophrenia studies included studies comparing groups with schizophrenia, to controls balanced for IQ, gender, and handedness were selected. Autistic Disorder studies included studies comparing groups with autism, or autism-spectrum conditions such as high-functioning autism (HFA) and Asperger, to controls. Our definition of autism is when the subject's IQ is below 70 and has a speech acquisition delay of 36 months. Subjects with HFA and Asperger have IQ above 70, and the latter has no delay in speech acquisition.

VBM studies of schizophrenia comprise a heterogeneous collection of samples recruited at varying stages of illness with variable medication exposure. Since antipsychotic medication has been clearly shown to affect brain volume even early in treatment [24], [25], we restricted our selection of VBM studies of schizophrenia to those with antipsychotic-naïve patients experiencing their first episode (FE) of schizophrenia, or patients who had been on treatment for less than 3 months. In addition, by excluding studies of patients with chronic schizophrenia, the mean age of patients in the schizophrenia studies sampled was closer to the ages in autism studies. The studies must have used voxel-based morphometry methods, and reported co-ordinates in 3D stereotactic space. Where data from an earlier study formed part of another study, the largest was included. One study (Meda et al., 2008) comprised of patients from multiple scanning sites. Only the data from Western Psychiatric Institute and Clinic at the University of Pittsburgh (WPIC) were included as all of these were mediation-naïve and FE. The selected studies are listed in table 1. [12], [16], [26], [27], [28], [29], [30], [31], [32], [33], [34], [35], [36], [37], [38], [39], [40], [41], [42], [43], [44], [45], [46], [47], [48].

**Table 1:** Voxel-based studies included in the meta-analysis.

Voxel-based Studies	Disorder Type	Mean	Global tissue	Sample Size		Mean Age	
		IQ	Difference	*Subjects*	*Controls*	*Subjects*	*Controls*
*Autism Spectrum Disorders*							
Abell et al., 1999	Asperger	>70	n/a	15	15	28.8	25
Boddaert et al., 2004	Autism	<70	n/a	21	12	9.3	10.8
Bonilha et al., 2008	Autism	<70	n/a	12	16	12.4	13.2
Brieber et al., 2007	HFA, Asperger	>70	No	15	15	14.2	13.3
Craig et al., 2007	HFA, Asperger	>70	No	14	19	37.9	35
Ecker et al., 2009	HFA	>70	No	22	22	27	28
Hyde et al., 2009	HFA	>70	No	15	13	22.7	19.2
Ke et al., 2008	HFA	>70	No	17	15	10	9.7
Kwon et al., 2004	HFA, Asperger	>70	n/a	20	13	14	13.6
McAlonan et al., 2002	Asperger	>70	No	21	24	32	33
McAlonan et al., 2008	HFA, Asperger	>70	No	33	55	11.4	10.7
Roja et al., 2006	HFA	>70	No	24	23	22.6	21.4
Toal et al., 2009	Autism, HFA	>70	No	65	33	31	32
Waiter et al., 2004	HFA, Asperger	>70	>GM	16	16	15.4	15.5
Wilson et al., 2009	HFA	>70	No	10	10	30	29.4
				**320**	**301**	**21.2**	**20.7**
*Schizophrenia*							
Chua et al., 2007	NN-FES	>70	<GM	26	38	32	33
Ebdrup et al. 2010	NN-FES	>70	No	29	43	25.7	26.9
Kasparek et al., 2007	NT-FES (7 weeks)	n/a	No	49	127	23.7	24.1
Lui et al., 2009	NN-FES	>70	n/a	68	68	24.7	24.7
Meda et al., 2008 (WPIC)	NN-FES	n/a	No	22	21	25	26.2
Molina et al. 2010	NT-FES (<1 week)	>70	No	30	40	25.8	29.4
Salgado-Pineda et al., 2003	NN-FES	n/a	No	13	13	23.8	23.4
Schaufelberger et al., 2007	NT-FES (<18 weeks)	>70	No	62	94	27.6	30.2
Venkatasubramanian, 2010	NN-FES	>70	<GM	30	27	30.1	27.4
Witthaus et al, 2009	NT-FES (<2 weeks)	>70	No	23	29	26.4	25.7
				**352**	**500**	**26.5**	**27.1**

Global tissue difference is any significant total grey matter difference compared to controls (*HFA, high-functioning autism; NN-FES, neuroleptic-naïve first episode schizophrenia patients; NT-FES, neuroleptic-treated first episode schizophrenia patients; GM grey, matter*).

### MNI to Talairach

Co-ordinates in Montreal Neurological Institute (MNI) format were transformed into Talairach using the “Lancaster transform”, icbm2tal. Co-ordinates transformed to Talairach space by using the Brett transformation, mni2tal, were transformed to the original MNI using Brett transformation, and reconverted to Talairach using icbm2tal [49], [50].

### Quantified Anatomical Likelihood Estimation (ALE) Approach

There were a total of 313 co-ordinates (197 for autistic disorders, 116 for first-episode schizophrenia) extracted from the studies listed in Table 1. Each condition's datasets were imputed into our in-house ALE kernel derived from open source software available at http://csl.georgetown.edu/software/ [20]. An individual ‘likelihood map’, which reflects the probability of finding grey matter differences, was generated for each study in the list, and smoothed with a 8 mm FWHM Gaussian kernel [23]. Likelihood maps from studies of the same condition were grouped together and averaged into a mean likelihood map of that condition. The mean map of ASD and schizophrenia was generated to prevent the condition with greatest number of foci reported biasing the final joint ALE result. The mean maps were summated to a joint likelihood map and 10,000 permutations used to sample the null distribution and test the probability that any given voxel of the joint likelihood map was significant. Results were thresholded at false-discovery rate p<0.05 and clusters greater than 100 mm^3^ were retained. Clusters generated from a single studies only were not reported in the resultant joint ALE. The ‘intensity’ ratio of the mean disorder maps to the final joint map at each resultant cluster was calculated. In this way the contribution of each condition to the final ALE result could be estimated (Matlab and SPM5 scripts available on request). For example, for a resultant cluster, high percentage contribution from one condition implied a high chance that foci from studies of that condition would be found therein. A 50% contribution from each condition suggested an equal chance that foci from either condition formed the cluster. Separate ALE analyses were conducted for grey matter deficits (188 foci) and excesses (133 foci).

## Results

### Global grey matter volume differences

Global grey matter volumes were generally found to be no different from control samples. The exceptions were 1 study of ASD reporting greater total GM in ASD than controls [46] and 2 studies of schizophrenia reporting less total GM than controls [30], [45] (see Table 1).

### Lower regional Grey Matter volumes

**Figure 1:**
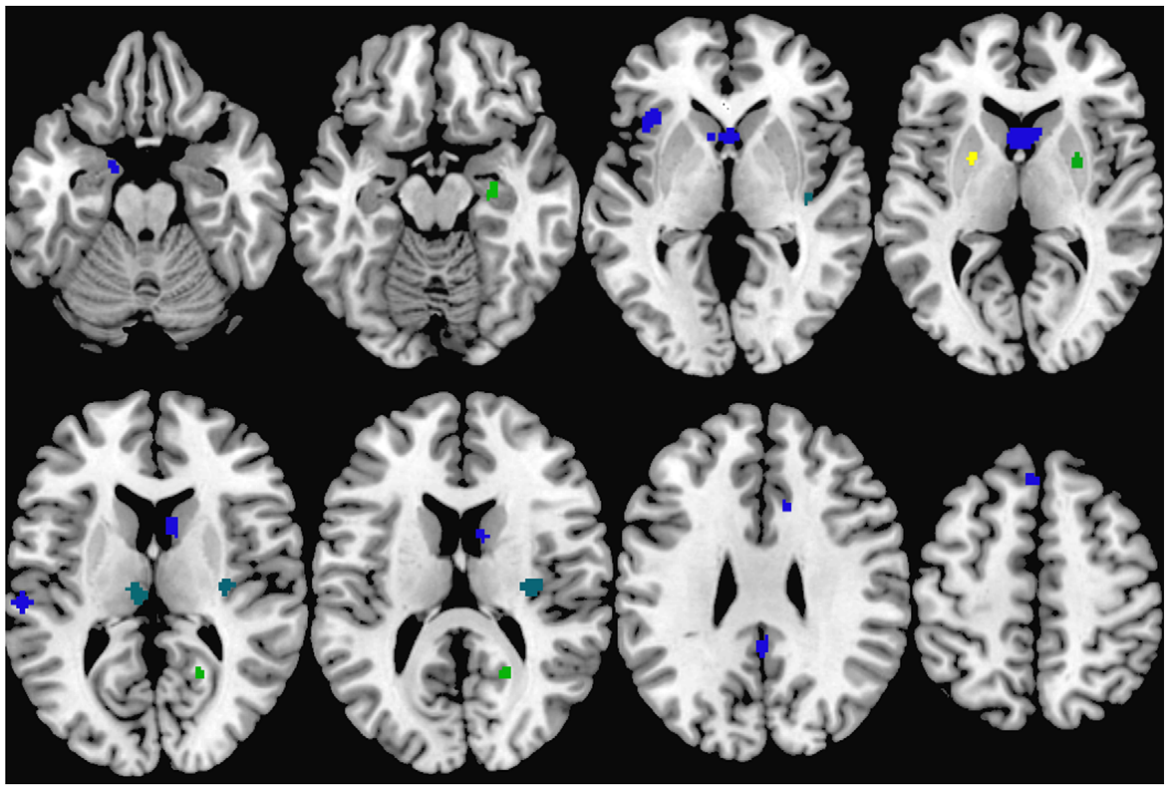
Lower grey matter volumes in ASD and Schizophrenia. Clusters indicating relationship between brain regions and condition are colour-coded as follows: blue for clusters contributed to mostly by schizophrenia studies, yellow for clusters contributed to mostly by ASD studies, and green for clusters contributed to by both conditions.

There were a total of 15 resultant ALE clusters showing lower grey matter volume compared to typical controls across schizophrenia-ASD studies. Brain structures that commonly affected by the two conditions included the right parahippocampal gyrus, posterior cingulate, putamen, insula and left thalamus. ‘Schizophrenia-only’ clusters were mostly located in the left hemisphere (left prefrontal cortex, insula, precuneus/cingulate, caudate, and amygdala) except for two regions (cingulate and middle frontal gyrus) in the right hemisphere. One cluster of lower grey matter volume in the left putamen was uniquely generated from ASD studies (please see Figure 1, Table 2).

**Table 2:** ALE Clusters formed in less grey matter.

Cluster	Cluster Center	Cluster Location	Cluster contributed by ASD studies (%)	Cluster contributed by Schizophrenia studies (%)
1	(−23,2,5)	Left Putamen	99.8	0.2
2	(28,−14,−15)	Right Parahippocampal Gyrus	42.9	57.1
3	(21,−56,14)	Right Posterior Cingulate (BA 30)	41.1	58.9
4	(28,0,6)	Right Putamen	38.9	61.1
5	(39,−20,−4)	Right Insula	23.1	76.9
6	(−7,−20,10)	Left Thalamus	23.1	76.9
7	(32,−17,15)	Right Insula	22.6	77.4
8	(0,−45,32)	Left Precuneus/Cingulate (BA 31)	0.4	99.6
9	(10,21,32)	Right Cingulate Gyrus (BA 32)	0.2	99.8
10	(−38,22, 0)	Left Insula/Inferior Frontal Gyrus	0.1	99.9
11	(−2,32,53)	Left Superior Frontal Gyrus (BA 8)	0.1	99.9
12	(2,12,6)	Left Caudate (Caudate Head/Body)	0	100
13	(−60,−24,12)	Left Temporal Gyrus (BA 42)	0	100
14	(−16,−2,−21)	Left Uncus/Amygdala (BA 34)	0	100
15	(43,33,21)	Right Middle Frontal Gyrus (BA 46)	0	100

### Grey Matter Excess

**Figure 2:**
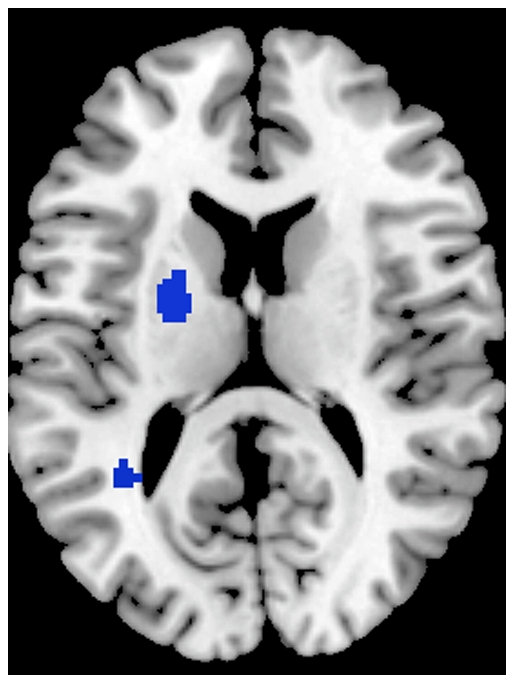
Greater grey matter volumes in ASD and Schizophrenia. Clusters indicating relationship between brain regions and condition are colour-coded as follows: blue for clusters contributed to mostly by schizophrenia studies, yellow for clusters contributed to mostly by ASD studies, and green for clusters contributed to by both conditions.

Only two ALE clusters showed grey matter volume enlargement. They were located in the left superior temporal gyrus and putamen and primarily generated by schizophrenia studies (shown in Figure 2, Table 3).

**Table 3:** ALE clusters formed in more grey matter.

Cluster	Cluster Center	Cluster Location	Cluster contributed by ASD studies (%)	Cluster contributed by Schizophrenia studies (%)
1	(−34,−50,6)	Left Superior Temporal Gyrus (BA 22)	7.8	92.2
2	(−22,0,12)	Left Putamen	5.6	94.4

## Discussion

Our study presents a novel means to examine brain structural similarities of 2 conditions. The growing number of VBM datasets available for ASD and schizophrenia meant that it was possible to try to balance the groups as much as possible in terms of IQ, age, drug treatment (schizophrenia samples were anti-psychotic-naïve), and total foci included. We describe a technique to calculate the percentage contribution of each condition to regional grey matter differences across the whole brain.

**Figure 3:**
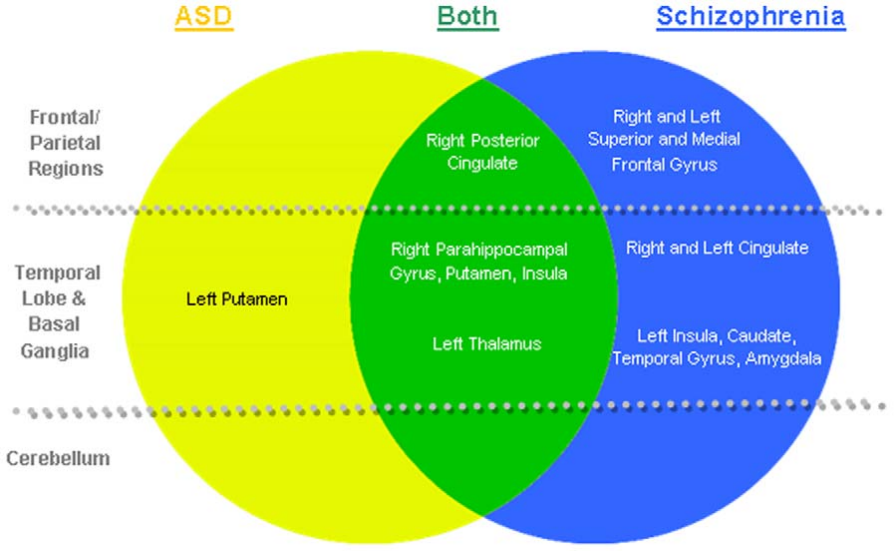
Distinct and overlapping regions of grey matter deficits found in ASD and Schizophrenia.

Our findings summarized in Figure 3, suggest that there are indeed considerable brain structural similarities between schizophrenia and ASD since both conditions result in lower grey matter in the right parahippocampal gyrus, posterior cingulate, putamen, clastrum and left thalamus. However a number of regions where lower grey matter volume was more specific to either condition were also identified. Lower regional grey matter volumes in the left hemispheric were extensive and highly schizophrenia-dependent apart from lower putamen volumes which were primarily driven by ASD.

This pattern of grey matter differences in schizophrenia and autism fits well with Middleton and Strick's prediction (2000) that abnormalities within basal ganglia loop systems may underlie neuropsychiatric symptoms [51], [52]. Basal ganglia loop systems are organized in a series of parallel but overlapping circuits through cortex - basal ganglia – thalamus - cortex which subserve motor and non-motor functions. Patients with damage to different parts of the circuit may therefore show similar symptoms. For example, Middleton and Strick highlighted observations that patients with pallidal lesions have cognitive deficits, obsessive-compulsive behaviors and even ‘psychic akinesia’ similar to neuropsychiatric conditions. Our results indicate that the limbic loop in particular, incorporating cingulate, striatum and thalamus [53], is affected by schizophrenia and ASD, and this may at least partly explain their shared socio-emotional symptoms.

Disruption within basal ganglia loop systems is also thought to explain impaired sensorimotor gating in both ASD [15], [16] and schizophrenia [18], [54], [55], [56]. Sensorimotor gating reflects the ability of an organism to filter out irrelevant stimuli. An operational measure of sensorimotor gating is made in the ‘prepulse inhibition of startle’ paradigm (PPI), whereby a subthreshold prepulse stimulus presented around 100 ms before a strong, startle-inducing stimulus, can attenuate the startle response. PPI impairment is a well-recognised endophenotypic trait for schizophrenia and ‘related’ disorders [57], [58]. Our results suggest that shared neuroanatomical characteristic are found in members of the ‘family of sensorimotor gating disorders’ [59].

Medial temporal lobe structures have long been postulated to play a role in autism and schizophrenia. In his seminal review in 1991, Delong conceived of autism as a ‘developmental syndrome of hippocampal dysfunction’ [60]. He specifically considered that the hippocampus, acting as a ‘mulitdimensional’ central processor, integrates contextual and motivational information to generate adaptive responses. A failure of the processor leads to symptoms in multiple domains including behaviour, language and emotion. A similar hypothesis of disruption of hippocampal development has lead to a useful animal model of schizophrenia in which neonatal hippocampal lesions have good face and construct validity as a model for schizophrenia [61], [62]. However we place these differences in the context of a more general cortico-striatal-thalamic loop pathology as described above, which the literature suggests may have developmental origins [63], [64].

Similarly the amygdala has been implicated in both ASD and schizophrenia. The role of the amygdala has been emphasized as central in autism [65], and in schizophrenia, Ellison-Wright's ALE analysis provided evidence supporting a reduction in the left uncus/amygdala with illness progression [66]. However, in the present analysis, schizophrenia rather than ASD studies contributed to the lower amygdala volume result. This was a left hemisphere effect and is therefore consistent with numerous reports suggesting left more than right amygdala involvement in schizophrenia [67], [68], [69]. Even in children of patients with schizophrenia, left amygdala volumes have a negative correlation with memory impairment [70]. In studies of autism and schizophrenia, functional imaging during emotion tasks reveals underactivation of the amygdala, pointing to some shared abnormalities in amygdala-based social processing in both conditions [65], [71]. Our results here indicate that structural differences, in terms of lower grey matter volume in the amygdala, are a feature of schizophrenia not ASD.

The present meta-analysis, showing overlapping grey matter abnormalities in brain regions in 2 conditions with shared behavioural traits, supports the position that schizophrenia and autism are related and not entirely polar opposites as proposed by Crespi and Badcock [72]. In the latter conceptualization autism and schizophrenia are said to have diametrically opposite phenotypes which include “a general pattern of constrained overgrowth” in autism, “whereas schizophrenia involves undergrowth”[72]. Clearly, the studies of these conditions in young adulthood argue against this finding with overlapping regional brain volume reductions observed. While it is true that autism is associated with brain overgrowth in early childhood, this pattern is largely gone by adolescence and adulthood [73]. Recent evidence now indicates some brain overgrowth in very young male children at high risk of schizophrenia [74], bringing the conditions closer in anatomical terms even in early childhood.

We emphasize that, although we interpret our findings as indicative of overlapping neuroanatomical phenotype, we do not imply that schizophrenia and autism constitute a common entity. Our study indicates a number of brain regions discretely affected by schizophrenia or autism. The result is largely consistent with the findings of Toal et al. 2009, in which the authors compared two autistic groups (with psychosis or without psychosis) separately to controls, and reported coincident lowering of grey matter in many brain areas in both groups [12]. One of the main differences in psychotic and non-psychotic groups with autism was that the former had lower grey matter volumes in the right insula. This fits with our observation that lower grey matter in the right insula is more prominent in schizophrenia than autism. The schizophrenia-dependent right insula differences also align closely with our previous analysis of multiple published datasets showing that that both predisposition to schizophrenia and progression of schizophrenia involves smaller right insula volumes [22]. We interpreted this latter work as evidence for a likely role of the insula in emotional difficulties in both high risk individuals and patients with clinical illness [22]. Lower volume in the left insula was also primarily schizophrenia-driven. This fits with observations from region-of-interest analysis indicating that significantly lower left insula volumes in schizophrenia correlate strongly with bizarre delusions [75].

The Toal et al (2009) study also found cerebellar volume differences in autistic groups with and without psychosis and controls [12]. In fact smaller volumes in the cerebellum were more extensive in the autistic group with psychosis. In our present synthesis of studies, we found no evidence supporting cerebellar grey matter differences in ASD or schizophrenia. This was somewhat unexpected. Autism is generally thought to involve cerebellar pathology [76], [77], [78], [79], [80]. However, the present analysis did not examine white matter, therefore we cannot rule out the possibility that cerebellar white matter anomalies are a feature of either condition as has previously been reported [30], [81], [82].

The major challenge for further study is to try to understand how shared genetic and environmental risk factors acting to elicit such similar grey matter deficits in autism and schizophrenia have quite different illness progression. There are very clear clinical distinctions to be made between the 2 disorders, not least of which lies in their developmental trajectories. Autism is evident in early childhood and is pervasive. Schizophrenia tends to present in late adolescence or early adulthood, and is relatively quiescent during childhood. Therefore one important potential confounder in the present study is that schizophrenia samples were slightly older than autism samples included in the analysis. Numbers of children presenting with childhood onset schizophrenia are limited, making it a practical challenge to study these groups. The available VBM evidence suggests that the brain structural phenotype in early onset schizophrenia is much the same as that reported in older patients [83]. In addition, the available evidence indicates that at least some aetiological risk factors are common to childhood and adult onset groups [84], [85].

### Limitations

In common with all meta-analytic approaches, a major limitation of our study is the ‘file drawer’ problem. That is, studies with negative findings are less likely to be written up and published. Even if there exist studies reporting no significant group differences, the ALE analysis method cannot take account of absent foci. Another possible limitation to the present analysis is that VBM methodology changes over time. Grey matter differences have been quantified in terms of intensity or modulated to yield volume measures. This difficulty for meta-analysis of VBM data has recently received attention [66], [86], but the modest number of studies included in the present analysis meant this could not be accounted for. An important limitation of meta-analyses of VBM studies is that there is no currently agreed format for reporting results. For example, a range of statistical criteria is used to report results. Sometimes the T-value for individual peak maxima is recorded, sometimes not. Many studies report corrected p values, others do not. Sample sizes are not always balanced and this can affect the power of a result. Additional concerns such as variations in the size of the smoothing kernel, threshold size of clusters reported and application of small volume correction potentially influence the results of different studies [21], [86], [87], [88]. We agree with others in the field that what is needed is more ‘rigorous standards of data reporting’ [86], [88].

In conclusion, we find an appreciable brain structural concordance between schizophrenia and autism. Specifically, lower volumes within the limbic basal ganglia loop system appear to be common to both schizophrenia and autism, while lower grey matter volume in the left putamen (autism) and left fronto-striatal-temporal regions (schizophrenia) appears to distinguish the conditions in terms of grey matter circuitry. Thus our results lend support to theories that schizophrenia and autism have important similarities [19], and detract somewhat from theories that predict they fall on diametrically opposite ends of a continuum [72]. Our findings should therefore encourage further exploration of potential shared aetiologies and better understanding of the mechanisms separating the 2 conditions.
